# Supplementary material for: Nutritional Status of Children with Cerebral Palsy in Gorkha, Nepal: Findings from the Nepal Cerebral Palsy Register
Source: Nutrients. 2021 Jul 25;13(8):2537. doi: 10.3390/nu13082537 (PMC8399945; doi:10.3390/nu13082537)
Supplement: Supplementary file 1 [file nutrients-13-02537-s001.zip › nutrients-1287395-supplementary.pdf]

Table S1: Relationship between GMFCS level, school attendance (among children aged ≥5 years) and nutritional status of children with CP in NCPR

**Indicator/Nutritional status**	**GMFCS level I-II, n=82**			**GMFCS level III-V, n=100**		
	School-aged children, n=67			School-aseg children, n=69		
	Attends school, n=40	Does not go to school, n=27	p value	Attends school, n=13	Does not go to school, n=56	p value[1]
Weight-for-age (WAZ), n	16[2]	8[2]	N/A	3[2]	19[2]	N/A
Normal, n (%)	11 (68.8)	4 (50.0)	0.41	2 (66.7)	3 (15.8)	0.12
Underweight, n (%)	5 (31.3)	4 (50.0)		1 (33.3)	16 (84.2)	
HAZ, n	39[3]	24[3]	N/A	11[3]	53[3]	N/A
Normal, n (%)	24 (61.5)	7 (29.2)	**0.01**	4 (36.4)	7 (13.6)	**0.08**
Stunted, n (%)	15 (38.5)	17 (70.8)		7 (63.6)	46 (86.4)	
BAZ, n	38[3]	19[3]	N/A	10[3]	46[3]	N/A
Normal, n (%)	31 (81.6)	13 (68.4)	0.32	8 (80.0)	24 (52.2)	0.16
Thin, n (%)	7 (18.4)	6 (31.6)		2 (20.0)	22 (47.8)	

[1] Fisher's exact test (1-sided); [2] This analysis was carried out for children aged between ≥6 years (average school going age of children in Nepal) and 10 year 1 month (above which WAZ is not considered to reflect nutritional status of children hence not calculated); [3]This analysis was carried out for children aged between ≥6 years (average school going age of children in Nepal) and above. Additionally, there were data missing for school attendance and GMFCS level.

Table S2: Predictors of underweight, stunting and thinness among children with CP in Gorkha, Nepal (Unadjusted analysis)

**Predictors**[1]	**Underweight (WAZ<-2SD), n=45**		**Stunting (HAZ<-2SD), n=109**		**Thin (BAZ<-2SD), n=48**	
	OR [95% CI]	p value	OR [95% CI]	p value	OR [95% CI]	p value
**Sex**						
Male	*Ref*		*Ref*		*Ref*	
Female	2.7 [1.0, 6.8]	**0.04**	2.2 [1.1, 4.5]	**0.03**	1.1 [0.5, 2.3]	0.75
**Monthly family income**						
1000-14999 (10-149.9)	1.3 [0.3, 5.6]	0.69	0.3 [0.1, 1.5]	0.16	1.0 [0.3, 3.1]	0.97
15000-29999 (150-299.9)	0.4 [0.1, 1.6]	0.19	0.2 [0.1, 1.0]	0.05	1.1 [0.3, 3.7]	0.84
30000 and above (≥300)	*Ref*		*Ref*		*Ref*	
**Attendance to mainstream school**						
Yes	*Ref*		*Ref*		*Ref*	
No	6.2 [1.7, 22.6]	**0.01**	5.7 [2.6, 12.8]	**<0.001**	3.3 [1.4, 7.9]	**0.01**
**Topography of CP**						
Mono/ Hemiplegia	*Ref*		*Ref*		*Ref*	
Diplegia	1.5 [0.3, 8.0]	0.62	2.3 [0.6, 8.3]	0.20	0.8 [0.2, 3.3]	0.73
Tri/ Quadriplegia	1.9 [0.6, 5.8]	0.26	4.4 [1.9, 10.4]	**0.001**	1.9 [0.8, 4.6]	0.12
**GMFCS level**						
I-II	*Ref*		*Ref*		*Ref*	
III-V	3.4 [1.4, 8.4]	**0.01**	4.0 [2.0, 8.0]	**<0.001**	2.4 [1.2, 5.0]	**0.02**
**MACS level**						
I-II	*Ref*		*Ref*		*Ref*	
III-V	2.2 [0.8, 6.2]	0.12	3.6 [1.6, 7.7]	**0.001**	2.8 [1.3, 5.9]	**0.01**
**CFCS level**						
I-II	*Ref*		*Ref*		*Ref*	
III-V	2.2 [0.9, 5.4]	0.08	2.2 [1.1, 4.3]	**0.02**	2.2 [1.1, 4.3]	**0.03**
**Viking speech level**						
I-II	*Ref*		*Ref*		*Ref*	
III-V	1.8 [0.6, 5.0]	0.26	2.4 [1.2, 4.8]	**0.02**	1.6 [0.7, 3.4]	0.23
**Intellectual impairment**						
No	*Ref*		*Ref*		*Ref*	
Yes	2.8 [1.0, 8.0]	0.06	2.6 [1.2, 5.7]	**0.02**	2.3 [0.9, 5.5]	0.07

[1] Variables that were found significant in cross tabulation (e.g. table 2 and 3) were included in unadjusted logistic model as potential predictors of undernutrition among children with CP in Gorkha, Nepal.
